# The dynamic balance of import and export of zinc in *Escherichia coli* suggests a heterogeneous population response to stress

**DOI:** 10.1098/rsif.2015.0069

**Published:** 2015-05-06

**Authors:** Hiroki Takahashi, Taku Oshima, Jon L. Hobman, Neil Doherty, Selina R. Clayton, Mudassar Iqbal, Philip J. Hill, Toru Tobe, Naotake Ogasawara, Shigehiko Kanaya, Dov J. Stekel

**Affiliations:** 1 Medical Mycology Research Center, Chiba University, 1–8–1 Inohana, Chuo-ku, Chiba, Chiba 260–8673, Japan; 2 Graduate School of Biological Science, Nara Institute of Science and Technology, 8916–5, Takayama, Ikoma, Nara 630–0192, Japan; 3 Graduate School of Information Science, Nara Institute of Science and Technology, 8916–5, Takayama, Ikoma, Nara 630–0192, Japan; 4 School of Biosciences, The University of Nottingham, Sutton Bonington Campus, Sutton Bonington, Loughborough LE12 5RD, UK; 5 Laboratory of Molecular Medical Microbiology, Department of Biomedical Informatics, Osaka University Graduate School of Medicine, 1–7 Yamadaoka, Suita, Osaka 565–0871, Japan

**Keywords:** zinc homeostasis, *Escherichia coli*, mathematical model, bet hedging, statistical inference, stress response

## Abstract

Zinc is essential for life, but toxic in excess. Thus all cells must control their internal zinc concentration. We used a systems approach, alternating rounds of experiments and models, to further elucidate the zinc control systems in *Escherichia coli*. We measured the response to zinc of the main specific zinc import and export systems in the wild-type, and a series of deletion mutant strains. We interpreted these data with a detailed mathematical model and Bayesian model fitting routines. There are three key findings: first, that alternate, non-inducible importers and exporters are important. Second, that an internal zinc reservoir is essential for maintaining the internal zinc concentration. Third, our data fitting led us to propose that the cells mount a heterogeneous response to zinc: some respond effectively, while others die or stop growing. In a further round of experiments, we demonstrated lower viable cell counts in the mutant strain tested exposed to excess zinc, consistent with this hypothesis. A stochastic model simulation demonstrated considerable fluctuations in the cellular levels of the ZntA exporter protein, reinforcing this proposal. We hypothesize that maintaining population heterogeneity could be a bet-hedging response allowing a population of cells to survive in varied and fluctuating environments.

## 1. Introduction

Zinc is an essential micronutrient for all forms of life and acts as a cofactor for all six Enzyme Commission classes [1–3]. However, at high levels, zinc is toxic to cells [4]. Thus the concentration of internal free zinc must be controlled.

In *Escherichia coli*, zinc can be imported by the high-affinity ABC-type zinc uptake system ZnuABC [5,6] which consists of three components: a periplasmic-binding protein, ZnuA; a membrane-spanning protein, ZnuB; and an ATPase, ZnuC [7]. The expression of *znuABC* is repressed by the zinc uptake regulator, Zur, which acts as a dimer, containing four zinc ions in its active repressor form [5,6,8]. In the presence of zinc, the active Zur dimer binds DNA at the *znuABC* promoter, competes with RNA polymerase for promoter occupancy, and consequently acts as a repressor.

Zinc can be exported by ZntA, a P-type ATPase [9]. Expression of *zntA* is activated by ZntR, a member of the MerR family of regulators [10,11]. In the absence of zinc, ZntR binds to DNA at the *zntA* promoter but does not activate expression of *zntA*. In the presence of zinc, ZntR is converted into a transcriptional activator, changing the DNA conformation of the *zntA* promoter leading to enhanced binding of RNA polymerase and transcriptional activation [12].

In addition to the high-affinity zinc uptake and export transporters ZnuABC and ZntA, *E. coli* possesses subsidiary zinc importers and exporters that exhibit lower affinities for zinc. ZupT, a member of the ZIP family of transporters [13] is a constitutively expressed importer that facilitates the uptake of a broad-range of metal ions with a slight preference for Zn^2+^ [14,15]. There are two other transporters that may participate in low-specificity zinc uptake: PitA, an inorganic phosphate transporter and MntH, an Mn^2+^/Fe^2+^ transporter of the Nramp superfamily [16,17]. Zinc export is also provided by the cation diffusion facilitator (CDF) ZitB. Transcription of *zitB* is directly inducible by zinc [18]. A further CDF transporter, YiiP has been implicated in zinc export, although its main substrate *in vivo* is Fe^2+^ [19,20].

Both Zur and ZntR manifest femtomolar sensitivity to zinc *in vitro* [8]. Therefore, it has been postulated that the concentration of internal free zinc is approximately femtomolar [21]. Subsequent measurements *in vivo* showed mean internal zinc concentrations mostly between 10 and 30 pM [22], although with considerable variability outside that range. In contrast, the total zinc quota in the cell has been reported by both groups as approximately 0.2 mM [8,22], approximately 2000 times higher than zinc concentration in low zinc media, and indicates efficient uptake and storage of zinc [8,22]. Much of the stored zinc is believed to be in ribosomes, in particular the ribosomal L31 protein [23–26]. Low molecular weight thiols also act as a zinc reservoir in *Bacillus subtilis* [27]. Therefore, we would expect that a zinc reservoir would play an important role in zinc dynamics and homeostasis.

To date, one mathematical model has been developed for the zinc regulatory system in *E. coli* [28]. The model was constructed to describe results from *in vitro* experiments analysing interactions between Zur and ZntR and DNA, and the induction of the *znuABC* and *zntA* promoters [8], and was successful in explaining these experimental data. However, this *in vitro* model does not consider zinc homeostasis in live cells, and so does not include *in vivo* processes, e.g. import and export of zinc through alternative transporters, or the binding of zinc to other proteins in the cell, accounting for the overwhelming majority of cytoplasmic zinc [8].

## 2. Aims of study

This study aims to improve our understanding of *in vivo* zinc homeostasis gene regulation, using an iterative ‘systems biology’ approach, consisting of alternating rounds of experimental and theoretical work. Our first aim was to identify the transcriptional responses of the main zinc transporters, ZnuABC and ZntA, to both genetic and chemical perturbations. Specifically, we have generated experimental data for the *in vivo* transcriptional activity of the *znuC* and *zntA* promoters in six strains: wild-type, *ΔznuCB*, *ΔzntA*, *Δzur*, *ΔzntR* and *ΔznuCBΔzntA*. Furthermore, we have quantified the responses of each of these promoters in each of the strains in detailed *in vivo* time course experiments following zinc stress.

Our second aim was to determine whether our existing knowledge of transcriptional regulation could explain the experimental data. We developed a new mathematical model for *in vivo* regulation of internal zinc levels by *E. coli*, using a set of ordinary differential equations (ODEs). We have integrated the model both with literature data and our newly derived experimental data using a Monte Carlo Markov chain approach [29,30]. This allowed us to evaluate model fits to the data and establish plausible ranges of parameter values, and so to evaluate the importance of alternative zinc transporter proteins and a zinc reservoir in explaining the available data on zinc regulation.

The process of fitting the mathematical model to the experimental data led to new questions that we had not anticipated. Specifically, we were forced to hypothesize that the higher levels of zinc stress that we used experimentally were partially toxic to the cells. This hypothesis was tested in a further round of experiments looking at the toxicity of zinc to *E. coli* cells at different zinc concentrations. This combination of model-driven hypothesis generation and experimental confirmation led to a further hypothesis that ZntA would exhibit heterogeneous expression. This was tested using a stochastic model of the system [31,32] that we simulated with the parameters obtained from the model fits to our experiments.

## 3. Results

### 3.1. Experimental characterization of transcription responses of zinc import and export proteins

Activities of the zinc-regulated *znuC* and *zntA* promoters were measured in the six strains studied: wild-type, *ΔznuCB*, *ΔzntA*, *Δzur*, *ΔzntR* and *ΔznuCBΔzntA* using a Lux reporter system (see Material and methods). The reporter was also tested on the *hns* promoter as zinc-independent control (electronic supplementary material, figure S1). Zinc concentration in the batch of LB used for these experiments was measured by ICP-MS as 12.2 μM. Excess zinc conditions include the addition of either 12.5 μM or 100 μM zinc to LB, giving total zinc concentrations of 24.7 μM and 112.2 μM, and are referred to as LB^12.5^ and LB^100^, respectively.

### 3.2. *In vivo* data in LB conditions

**Figure 1.**
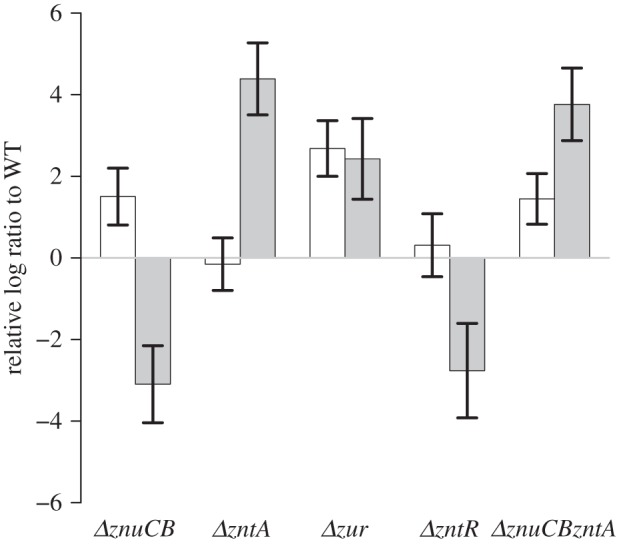
Induction of the P*znuCB* and P*zntA* in the five mutant strains relative to wild-type (log to base 2) in LB conditions. White and grey bars correspond to P*znuCB* and P*zntA*, respectively. Error bars indicate s.d.

Altered promoter activity was observed in the strains studied under LB conditions (figure 1). The highest activity of the *znuCB* promoter (P*znuCB*) was seen in the *Δzur* strain, concordant with Zur being a repressor of *znuABC* expression [5]. Induction was also seen in the *ΔznuCB* strain, also expected, as less zinc import should lead to derepression of P*znuC* by Zur; a similar argument holds for the *ΔznuCBΔzntA* strain.

The *zntA* promoter (P*zntA*) was strongly induced in the *Δzur* and *ΔzntA* strains. This is to be expected, as we would anticipate a rise in internal zinc concentrations as either zinc import by ZnuABC was derepressed or zinc export by ZntA was abolished. Expression of P*zntA* was greatly reduced in the *ΔzntR* strain, also expected as ZntR is the activator for *zntA* [10]. There is a similar reduction of *zntA* expression in the *ΔznuCB* strain, also expected, as there would be less zinc in the cell. The *ΔznuCBΔzntA* double mutant gave surprising results. *A priori* there are two possible outcomes: either the internal zinc concentration might decrease, as in the *ΔznuCB* strain, in which case we would expect P*znuCB* activity to increase and P*zntA* activity to decrease; or the internal zinc concentration might increase, as in the *ΔzntA* strain, in which case we would expect little change in P*znuCB* activity and an increase in P*zntA* activity. Instead, we observe increased activity of both promoters which appears to be incompatible with either scenario. The model provides an explanation for this phenomenon, as described below.

### 3.3. Time course of *in vivo* promoter activity following addition of zinc

**Figure 2.**
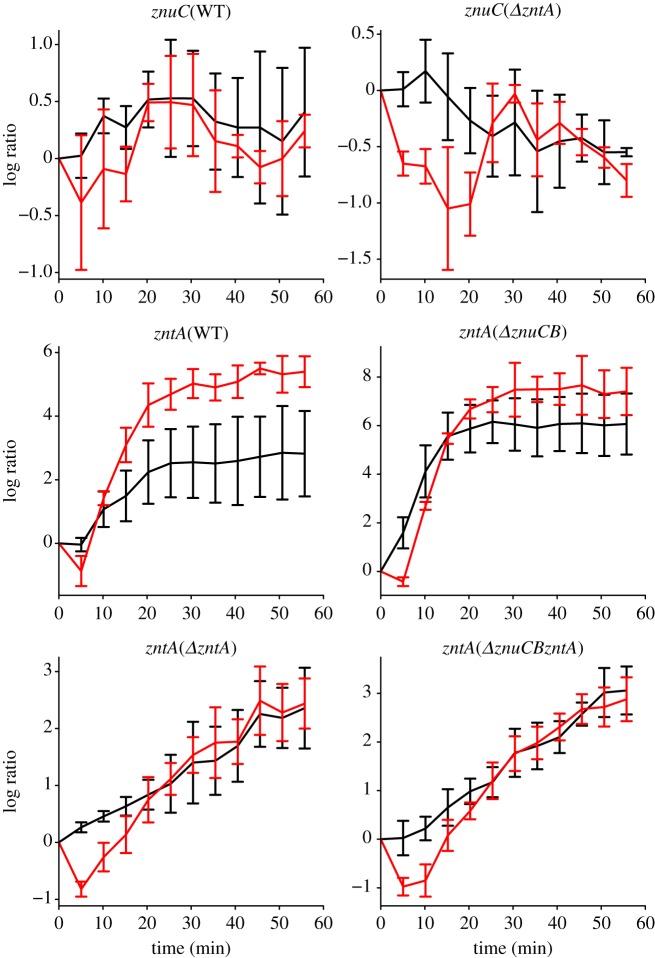
Time series of induction of the *znuC* and *zntA* promoters following addition of 12.5 μM Zn^2+^ (black lines) and 100 μM Zn^2+^ (red lines), log to base 2 ratios normalized to the value at *t* = 0. Plots show the wild-type responses and selected responses from other strains that show the most marked changes in expression (the remaining responses are plotted in electronic supplementary material, figure S1). Note that in all of the plots, there is an initial decrease in the reporter expression under 100 μM zinc conditions: we propose that this results from the partial lethality of these conditions. Note also that the level of induction of P*zntA* in the WT strain is different under the two different conditions, whereas in the other strains, the level of induction reaches a similar level once the initial dip is reversed. Error bars indicate s.d.

Figure 2 shows detailed time courses describing the activities of P*znuCB* and P*zntA* in the WT strain along with the most prominent responses in the mutant strains in LB^12.5^ and LB^100^; the less prominent responses are shown in electronic supplementary material, figure S2. In the WT, P*znuCB* shows little change in either condition, whereas P*zntA* shows marked increases in expression in both LB^12.5^ and LB^100^. A common feature of these and other responses is an initial decrease in induction in LB^100^. The induction of P*zntA* in LB^100^ (WT) and both LB^12.5^ and LB^100^ (*ΔznuCB* strain) were much larger (more than 4 [log_2_ ratio]) and faster (less than 30 min) than in the *ΔzntA* and *ΔznuCBΔzntA* strains in both LB^12.5^ and LB^100^. Although the induction of P*zntA* in the WT and *ΔznuCB* strains plateaued after 30 min following addition of zinc, the induction of P*zntA* in the *ΔzntA* and *ΔznuCBΔzntA* strains continued after addition of zinc until 50 min*.* Furthermore, in the *ΔzntA* strain, P*znuCB* showed a small decrease in induction. These observations suggest that there may be mechanisms to import external zinc into cells without ZnuABC and that ZntA is important to maintain the steady level of free zinc in *E. coli* cells. Interestingly, while the WT strain shows a clear difference in induction under the two concentrations of added zinc, in the mutant strains, the level of induction is similar in LB^12.5^ and LB^100^ (apart from the initial dip in promoter activity under the higher zinc concentration). The responses of the induced promoters are also considerably stronger than those observed in the *hns* promoter under LB^100^ conditions (electronic supplementary material, figure S1*b*) and so can be attributed to zinc-associated transcriptional change.

### 3.4. A new mathematical model for *in vivo* zinc transport and homeostasis

**Figure 3.**
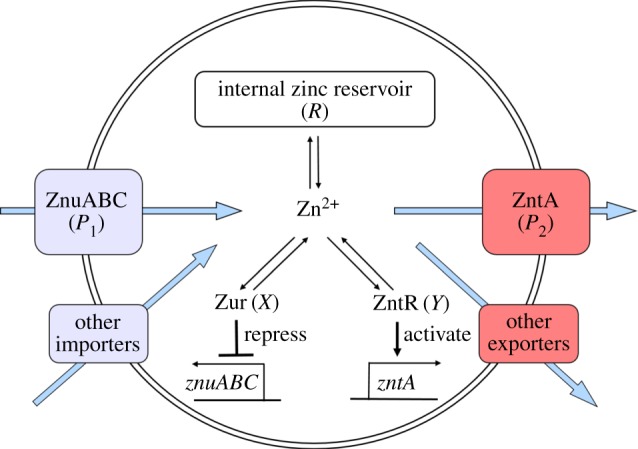
Cartoon of the mathematical model for the zinc regulation system in *E. coli*. Zinc import is both through the Zur-repressed ZnuABC system and through other importers. Zinc export is both through the ZntR-activated ZntA system and through other exporters. Ionic zinc can be bound and be released from the internal zinc reservoir; it also can bind to the Zur and ZntR proteins leading to their active forms as transcription factors.

We developed a mathematical model that describes the molecular processes of zinc homeostasis, in order to explain these experimental data along with literature data (figure 3). There are six variables: the concentration of ZnuABC (*P*_1_), the concentration of ZntA (*P*_2_), the concentration of active (zinc-bound) Zur (*X*), the concentration of active (zinc-bound) ZntR (*Y*), the concentration of zinc bound to ‘reservoir’ molecules, i.e. any other zinc-binding molecules in the cell (*R*) and the concentration of free (ionic) zinc in the cytoplasm (*z*). The equations are3.1
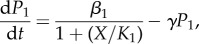
3.2
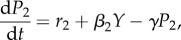
3.3
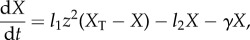
3.4
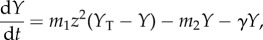
3.5
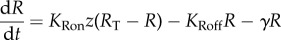
3.6
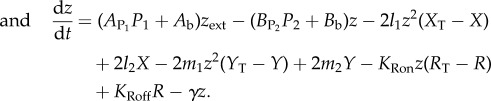

ZnuABC is produced at maximal rate *β*_1_ and is repressed by active Zur with Michaelis constant *K*_1_ (equation (3.1)). ZnuABC and all other modelled components are diluted due to cell growth at rate *γ*; the six strains examined could potentially grow at different rates so the value of *γ* is strain-dependent. ZntA is produced at a basal rate *r*_2_ and is activated by active ZntR with a constant of proportionality *β*_2_ (equation (3.2)). In the equation for active Zur, *X*_T_ represents the total amount of Zur in the system (assumed to be constant), and so inactive Zur is given by *X*_T_ − *X*. The binding of two zinc ions is required to convert inactive Zur to active Zur [6], at rate *l*_1_. The active form can also revert to the inactive form at rate *l*_2_ (equation (3.3)). Similarly, the binding of two zinc ions is required to convert inactive ZntR to active ZntR [10,12,33] at rate *m*_1_ and the reversion of active ZntR to its inactive form happens at rate *m*_2_ (equation (3.4)). In the equation for the zinc reservoir (equation (3.5)), the total reservoir size is *R*_T_ and so *R*_T_ − *R* represents the number of available zinc-binding sites. Zinc binds to free reservoir binding sites with mass action kinetics with rate *K*_Ron_ and dissociates with rate *K*_Roff_. The reservoir molecules are replenished to balance dilution due to cell growth so that the overall concentration of reservoir molecules remains constant. Import of ionic zinc is proportional to the concentration of external zinc (equation (3.6)), with basal rate *A*_b_ and linear dependence on ZnuABC with parameter *A*_P1_. Export is proportional to internal free zinc, with basal rate *B*_b_ and linear dependence on ZntA with parameter *B*_P2_. The remaining terms in equation (3.6), for the interactions of zinc ions with Zur, ZntR and the reservoir, have already been described.

### 3.5. Model fitting to data

Central to this work is the fitting of the mathematical model to the experimental data. This has been accomplished using the Metropolis–Hastings algorithm for parameter inference (see Material and methods). Convergence plots from the simulations and posterior distributions for all parameters are shown in electronic supplementary material, figure S3. Point estimates and ranges for all parameter values are shown in electronic supplementary material, table S1.

### 3.6. The model fits published *in vitro* data and data under LB conditions

**Figure 4.**
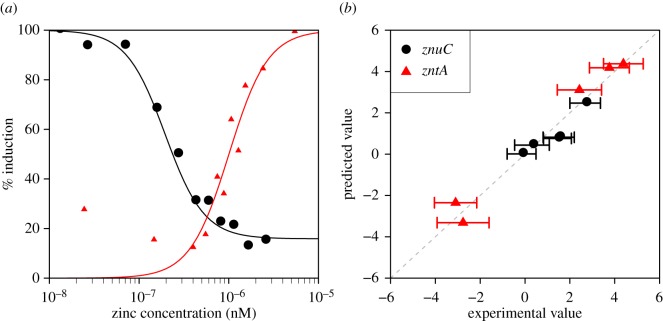
(*a*) Model fit to promoter induction data from [8] for the *znuC* and *zntA* promoters. The fit to data is extremely close and comparable to fits from previously published work [28]. (*b*) Model fit to the differential gene expression of the two promoters in each of the five mutant strains (log to base 2) that was reported in figure 1. There is an extremely strong concordance between the experimental data and the model values.

The model fit to the published zinc induction data [8] is good (figure 4*a*) and comparable with the fit of the previously published zinc model [28]. The model fit to our data under LB conditions is also excellent (figure 4*b*). In accordance with the experimental data, the model predicts that the highest induction of P*znuCB* was seen in the *Δzur* strain. Induction was also seen in the *ΔznuCB* and *ΔznuCBΔzntA* strains. Similarly, there is condordance between the experimental data and the model for P*zntA*, with strong induction in the *ΔzntA*, *Δzur* and *ΔznuCBΔzntA* strains, and strong repression in the *ΔznuCB* and *ΔzntR* strains.

Interestingly, the model predicts a reasonable fit to the increased induction of both P*znuCB* and P*zntA* in the double mutant: the experimentally observed fold differences relative to the WT are 2.7 and 13.6 for the two promoters, respectively, while the model fitted fold differences are 1.7 and 18.1. The fit is achieved because the growth rate of the double mutant is predicted to be lower than that of the WT (doubling times of 110 and 64 min, respectively) leading to an apparent increase in protein expression (and by association Lux protein expression). If instead one compares only the protein synthesis terms of equations (3.1) and (3.2), it is found that P*znuCB* has almost the same induction (0.99-fold increased), while P*zntA* is 10.6-fold increased. Thus the double mutant is behaving similarly to the *zntA* mutant, with little change in Zur repression and substantial increase in ZntR activation.

The steady-state concentration of free zinc of WT cells in LB predicted by the model is 2.9 pM (calculated from equation (3.6) with parameters from electronic supplementary material, table S1); this is in closer accordance with the measurements of Wang *et al.* [22] than the predictions of Outten & O'Halloran [8]. This concentration is a dynamic equilibrium, with high predicted flux of 44 nMs^−1^ through the free zinc compartment. Most of this flux is a balance between zinc ions imported into the cells and absorption of those ions into the zinc reservoir.

### 3.7. The model can fit LB^12.5^ zinc induction *in vivo* time course data

**Figure 5.**
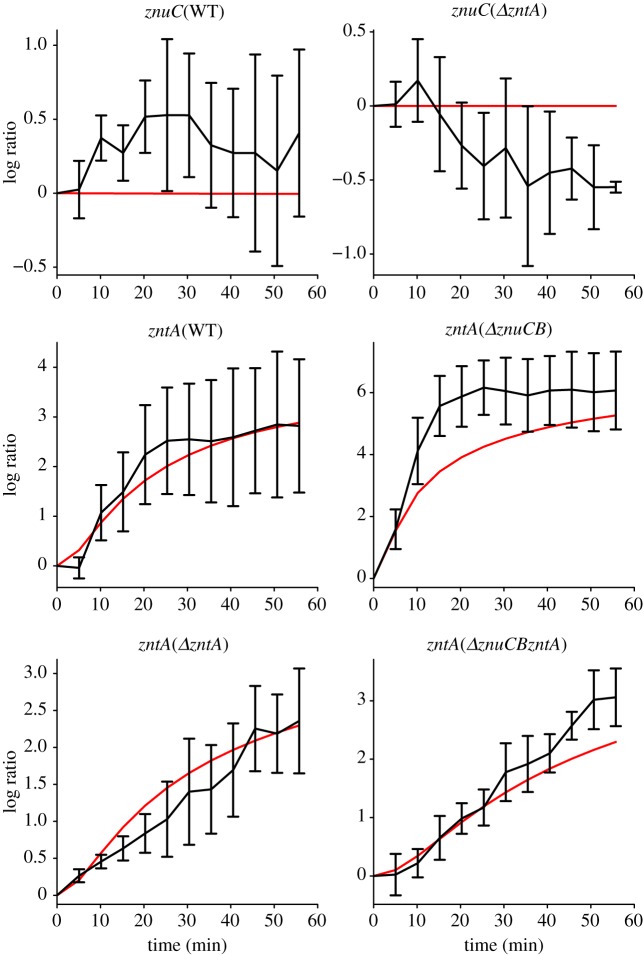
Log (to base 2) of expression relative to time *t* = 0 of induction data following addition of 12.5 μM Zn^2+^ for the same strain/promoter combinations as figure 1 (the remaining model fits are shown in electronic supplementary material, figure S4). Black line is experimental data; red line is model fit using best fit parameters from electronic supplementary material, table S1. The model fits every time series, matching both the timescale and degree of differential expression.

The model is able to fit all of the *in vivo* time course data for LB^12.5^ (figure 5 and electronic supplementary material, figure S4). In almost all cases, the model faithfully reproduces the experimental results, mostly fitting within the error bars (1 s.d.) of the data. The least good fit is to *PznuC* in the *ΔzntA* strain, where the model predicts little change, and experimentally there is a small decrease in expression (0.7-fold over the time course). The other interesting case is *PzntA* in the *ΔznuCB* strain, which shows a similar level of induction after 60 min, but on a slower timescale in the model compared with the experimental data. The goodness of fit can be quantified using *R*^2^ values associated with the LB^12.5^ time course data as a measure of the percentage of variability explained: these model fits have an *R*^2^ value of 92%. Overall, these results give considerable confidence in the model processes and parameter estimations.

### 3.8. Importance of basal import, basal export and reservoir to model fitting

Our model differs from that proposed by Cui *et al.* [28] in that it includes, most importantly: (i) the basal import of zinc, (ii) the basal export of zinc, and (iii) the zinc reservoir. The parameters associated with these processes have all been found to be important in explaining the data. For the basal import rate *A*_b_, the median value was 1.86 × 10^−3^ s^−1^; for the basal export rate *B*_b_, the median value was 2.63 × 10^2^ s^−1^; for the reservoir size *R*_T_, the median value was 1.97 × 10^5^ nM; and for the on-rate to the reservoir *K*_Ron_, the median value was 1.25 × 10^2^ nMs^−1^. The ranges of all four parameter values do not include zero (electronic supplementary material, table S1). Graphs for all four posterior distributions are shown in electronic supplementary material, figure S2. If basal import and export are excluded from the model, then the model cannot fit the data, with *R*^2^ values reduced from 92 to 9.6% (simulation results not shown).

The importance of basal import and export of zinc can also be highlighted by considering the proportion of zinc imported and exported by the basal and zinc-induced systems in LB and LB^12.5^ conditions (table 1). Using the median estimated parameters from the model, we predict that, in LB, 59% of external zinc is imported by basal importers and 25% of internal zinc is exported by basal exporters. In LB^12.5^, the proportion of import by basal exporters remains approximately the same, while the increase in ZntA expression leads to a reduction of the proportion of zinc exported by basal exporters to just 4.8%. The importance of the reservoir can be further highlighted by the calculation that the overwhelming majority of the internal zinc is predicted to be in the zinc reservoir (the ratio of the concentrations of free zinc to reservoir zinc is approx. 3.9 × 10^−8^), in agreement with published data [8].

**Table 1.** Percentage of zinc trafficked by basal systems. The proportion of zinc imported and exported through the basal systems as opposed to the zinc-induced systems has been calculated for the WT strains under LB and 12.5 μM added zinc conditions. The majority of zinc is predicted to be trafficked through the zinc-dependent ZnuABC and ZntA proteins, but a sizeable proportion is predicted to be trafficked through the basal (non-specific) systems. In the zinc added system, there is very little predicted change in ZnuABC expression, and so the proportion of zinc basally imported is hardly changed. In contrast, there is a considerable increase in ZntA expression, and so the proportion of zinc exported through ZntA increases from 75 to over 95%.

condition	*A*_b_	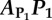	% zinc basal import	*B*_b_	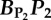	% zinc basal export
LB	1.86 × 10^−3^	1.28 × 10^−3^	59	2.63 × 10^2^	7.72 × 10^2^	25
+12.5 µM Zn^2+^	1.86 × 10^−3^	1.27 × 10^−3^	59	2.63 × 10^2^	5.17 × 10^3^	4.8

### 3.9. Impossibility of fitting model to *in vivo* data in LB^100^ conditions

**Figure 6.**
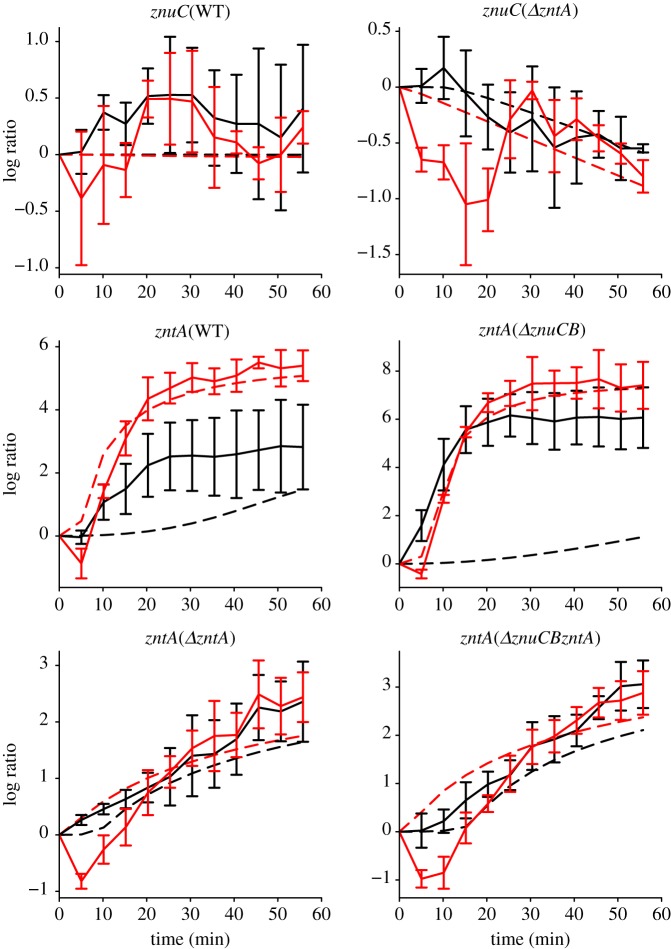
Log (to base 2) of expression relative to time *t* = 0 of induction data following addition of both 12.5 μM Zn^2+^ and 100 μM Zn^2+^ for the same strain/promoter combinations as figure 1 (the remaining model fits are shown in electronic supplementary material, figure S5). Some of the model fits are very poor, especially for P*zntA* in the WT and *ΔznuCB* strains*.* In comparing the goodness of fits, it is important to note that the scales of the *y*-axes are very different in different plots, so that the worst fit (P*zntA* in *ΔznuCB*) is associated with the data showing the greatest level of promoter induction. Black and red dashed lines correspond to model fits using best fit parameters about 12.5 μM Zn^2+^ and 100 μM Zn^2+^, respectively.

We were unable to fit the model to all of the data when we included time-series data for LB^100^ (figure 6 and electronic supplementary material, figure S5). While much of the data can be fitted, the model cannot fit the data in the two cases that demonstrate the highest levels of induction: P*zntA* in the WT and *ΔznuCB* strains. In both cases, the model can fit the LB^100^ conditions at the expense of the LB^12.5^ conditions, where the model predicts very little induction. As a consequence, the *R*^2^ value is reduced from 92 to 30%. The poor model fit when the LB^100^ data are included is not confined to the particular form of the ODE model presented in this manuscript: a wide range of different models were trialled, including models with saturating terms for zinc import, and they all displayed similar or worse patterns of behaviour (data not shown).

In order to explain the lack of model fit, we hypothesized that the behaviour of the cells in LB^100^ is different from their behaviour in LB or in LB^12.5^. Specifically, our hypothesis is that in LB^100^, the population response is heterogeneous, with some cells killed by the zinc stress, and only those cells with a strong zinc export phenotype surviving. In this case, ODE models, that describe the average behaviour of a homogeneous population, could never fit these data. This hypothesis is consistent with the dip in light output seen in the majority of cases in LB^100^ (figure 2 and electronic supplementary material, figure S1), as it is plausible that that dip could be associated with cell killing. (The luminescence assays measure light output from the whole population, so a reduction in light output is consistent either with a lower overall level of light output in a homogeneous population, or with a heterogeneous population in which a proportion is no longer producing light.)

This leads to two testable predictions. First, we expect a degree of cell death in LB^100^ that would not be seen in LB or LB^12.5^. Second, we expect that a stochastic version of the model, which is able to describe the random variability in individual cells, would display a high level of variability in ZntA expression. When 100 μM of zinc is added, those cells expressing high levels of ZntA might survive, whereas those cells expressing low levels of ZntA might be killed. Sections 3.10 and 3.11 describe results associated with testing these hypotheses.

### 3.10. Lower viable cell counts under LB^100^ conditions

**Figure 7.**
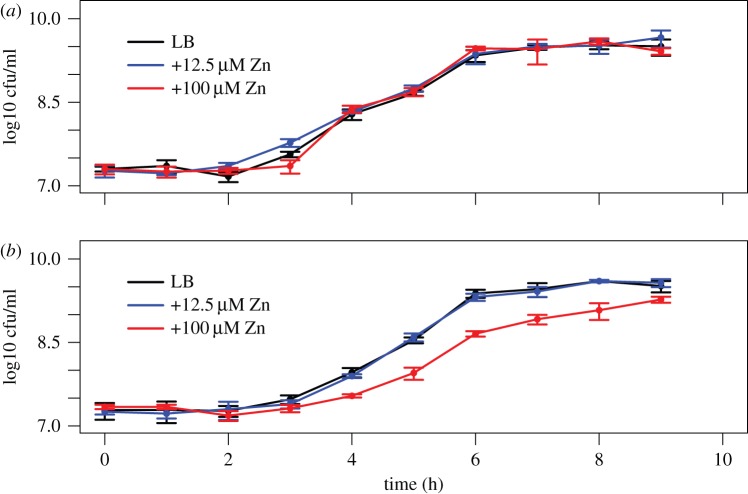
Viable cell counts for (*a*) WT and (*b*) double mutant strains grown in either LB, LB + 12.5 μM zinc or LB + 100 μM zinc. Error bars are 1 s.d. from triplicate experiments. In all conditions, a classical growth curve is seen, displaying lag, exponential and stationary phases. The WT strain shows little difference between the three conditions. The double mutant shows little difference between LB and 12.5 μM added zinc, but the viable cell counts are considerably reduced under 100 μM added zinc conditions. This result is consistent with the hypothesis that under strong zinc stress, there is a heterogeneous response in the population, with some cells growing, and others either dying or entering a non-growing state. Thus these results are experimental confirmation of the hypothesis put forward as a consequence of the mathematical model.

The WT and double mutant strains were grown in LB, LB^12.5^ and LB^100^, and the viable cell count assessed as a function of time (figure 7). The WT strain shows little difference under the three conditions studied. In contrast, the double mutant shows no difference between LB and LB^12.5^, but a considerably decreased viable cell count in LB^100^. The greatest difference, a reduction of over 80%, is seen at 6 h, with 2.4 × 10^9^ cfu ml^−1^ in LB but only 4.53 × 10^8^ cfu ml^−1^ in LB^100^. This result is consistent with the population heterogeneity hypothesis, of some cells growing, and other cells either dying or entering a non-growing state.

### 3.11. Prediction of stochasticity of ZntA protein levels in single cells from a stochastic model

In order to explore the ZnuABC and ZntA protein levels in single cells, a stochastic model was developed. This model contains exactly the same processes as the ODE model described above, but uses a discrete chemical reaction scheme to describe them [31], and thus captures intrinsic variability due to molecular events [34]. The complete chemical reaction scheme is given in the supporting SBML file (zinc.xml).

**Figure 8.**
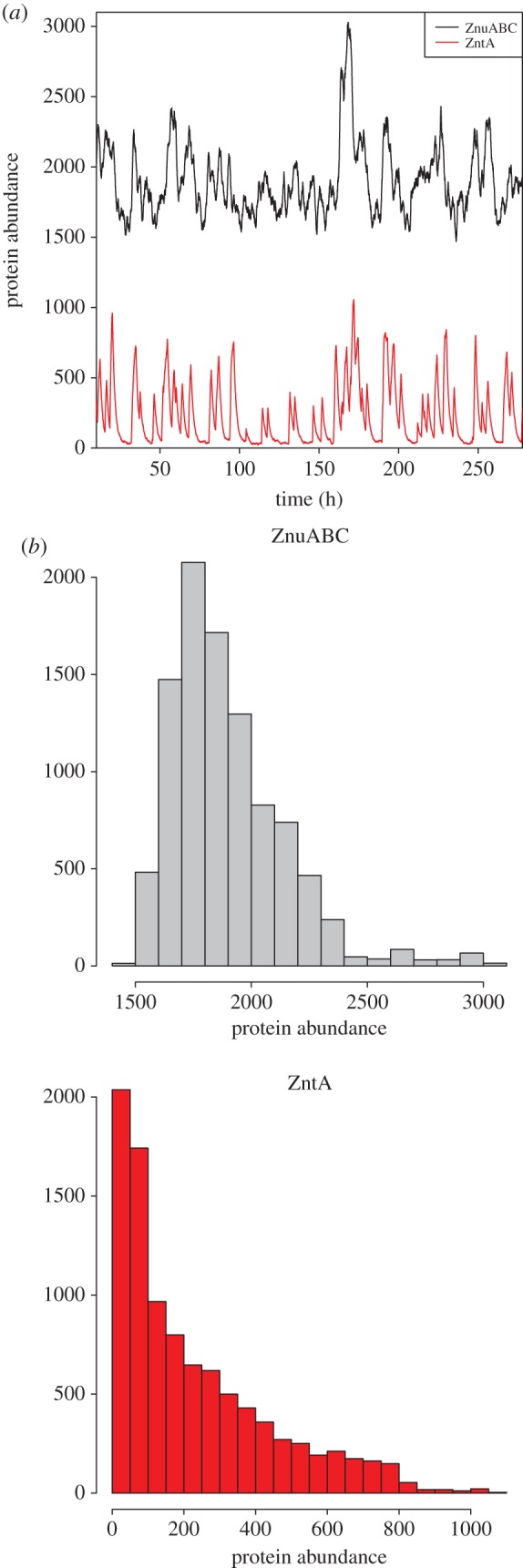
(*a*) Realization of a stochastic simulation showing ZnuC and ZntA protein abundance. There are small fluctuations in ZnuC abundance around a mean consistent with Poisson noise. In contrast, there are very large fluctuations in ZntA abundance, consistent with bursty production of protein. The timescale of the simulation is considerably longer than a cell cycle so the fluctuations are representative of the variability that would be seen in a population of cells. (*b*) Corresponding histograms of protein expression of ZnuC and (*c*) ZntA showing that ZnuC has a relatively tight distribution (mean 1943, s.d. 434, coefficient of variability 22%), while ZntA has a very broad distribution (mean 268, s.d. 324, coefficient of variability 121%).

Outputs from an example simulation are shown in figure 8. The ZnuABC and ZntA protein levels display very different behaviours. ZnuABC rises to a steady state of approximately 1943 molecules per cell, and then shows fluctuations around this steady state with a standard deviation of approximately 434 (coefficient of variability 22%), which is greater than Poisson noise, but consistent with negative regulation [35]. In contrast, the ZntA curve shows much greater variability, with a mean of 268 molecules per cell and s.d. of 324 (coefficient of variability 121%), and bursty expression leading to sharp peaks followed by dilution to low numbers of proteins per cell. This pattern occurs on a timescale considerably slower than the cell cycle; thus individual cells in a population would contain different levels of ZntA, with some cells having high expression and other cells with low expression. This confirms our second prediction.

The difference observed in the model in the variabilities of ZnuABC and ZntA arises from the actions of their regulators. In LB, the majority of Zur molecules are bound by zinc, while the majority of ZntR molecules are unbound by zinc. Zur is a repressor in this form, and P*znuCB* is usually bound by the Zur–zinc complex. The fluctuations in ZnuABC production arise due to the occasional unbinding of Zur followed by binding of another Zur dimer. These events are relatively common and are largely independent of internal free zinc. In contrast, ZntR is an activator when bound by zinc. P*zntA* is usually not bound by ZntR, and ZntA is only produced when a ZntR–zinc complex is formed and binds to P*zntA*. Because of the scarcity of free (ionic) zinc, these events are much rarer, and when they do occur, a large burst of protein is produced. ZntA production is also sensitive to stochasticity in free zinc abundance.

## 4. Discussion

The aim of this work is to develop a detailed understanding of the responses of the zinc homeostasis system in *E. coli* K-12 to added zinc, with a specific focus on the Zur-regulated importer system ZnuABC and the ZntR-regulated exporter ZntA. We have generated novel experimental data on six strains, with different key zinc-regulated genes deleted, including time course transcriptional responses of zinc uptake and efflux genes to added zinc. These data, along with data derived by previous groups, have been integrated with a detailed, mechanistic, mathematical model, using a Monte Carlo approach to fit this model to the data. As part of the model fitting, we could only fit data with the smaller concentration of added zinc (12.5 μM) and we could not use the data from the higher concentration of added zinc (100 μM). This led us to the hypothesis that under higher zinc concentrations, the cells demonstrate a heterogeneous response to zinc stress: some cells either do not grow or die, whereas others mount a successful response.

We tested this hypothesis both experimentally, by measuring viable cell counts, and theoretically, by constructing a stochastic model for the system in order to assess the level of heterogeneity in the cell responses. Both these approaches confirmed our hypothesis: in experiments, the viable cell counts were lower with 100 μM added zinc for the double mutant strain; and theoretically we have shown that the cells display considerable heterogeneity of expression of the ZntA exporter even under ‘normal’ conditions. While the lower viable cell counts could potentially be attributed to slower growth of a homogeneous population, such an interpretation is incompatible both with the analysis of the time course data and with the output of the stochastic model.

These results highlight the value of experimental evaluation of the heterogeneity of the zinc response in a population of cells, for example by using single cell assays with fluorescent reporters and flow cytometry. Stochastic models describing population heterogeneity could be calibrated against such data. These experiments would overcome the potential ambiguity of the viable cell counts. Moreover, although we have reported stochastic fluctuations of the ZntA protein, we propose that the double mutant, which cannot produce ZntA, itself has a heterogeneous population response. Therefore, we anticipate that other relevant proteins are expressed heterogeneously, possibly including the non-specific exporters. Future stochastic models could investigate such heterogeneity and could be meaningfully constructed once detailed population level data from single cell assays are available.

There are three main conclusions to be drawn from this research. First, the model shows that zinc import and export is not just mediated by the specific inducible or repressible Zur/ZnuABC and ZntR/ZntA systems, but also by non-specific transporters, in concordance with previously published experimental work. These include the alternative lower specificity Zn^2+^ importers ZupT, PitA and MntH [14,16,17] and alternative lower specificity zinc exporters ZitB [18] and YiiP [19,20]. This is likely to result from the fundamental chemistry of transition metal ions: biological import and export systems appear to lack recognition specificity between metals such as Zn, Cd and Pb. Thus, many transition metal import and export systems are likely to be cross-reactive, e.g. ZntA has been reported to be able to export Cd^2+^ and Pb^2+^ under certain conditions as well as Zn^2+^ [36]. Thus the net phenotype to ensure uptake of essential metals but removal of toxins will depend on the totality of import and export systems in a cell.

Second, the model shows the importance of the zinc reservoir, that contains the vast majority of zinc in the cell [8,22]. Previous mathematical models have not included this, but the reservoir is necessary for the cell for two reasons: (i) for the functional mobilization of zinc by turnover of L31, liberating zinc for other essential uses in times of zinc depletion, and replacement of L31 by the paralog YkgM protein which lacks zinc; and (ii) the reservoir acts as a buffer to protect against zinc toxicity from free zinc in the cell.

Third is the proposed heterogenous expression of ZntA and response of the population to zinc stress. Heterogeneous gene expression has been suggested as a mechanism for ‘bet-hedging’: greater overall clonal fitness because a sub-population is primed to respond to potential lethal stress [37,38]. Our results suggest that this might be the case for the zinc export system in *E. coli*. Given the cross-reactivity of metals, we speculate that it could be the case for other metal export systems too, leading to a population of cells with sub-populations capable of responding to a wide range of environmental stresses. Such speculation would require further experimental work [39].

The model also makes predictions relevant to the concentration of internal free zinc. Previous studies have estimated this to be in the femtomolar range [8], whereas other studies have measured it in the picomolar range [22]. It has been argued [22] that the discepency between the femtomolar sensitivity of Zur and ZntR and the picomolar internal zinc pool may arise because the zinc occupancy of these transcription factors does not rapidly equilibriate [22]. Our model estimates picomolar internal free zinc concentration, which is more consistent with the measurements made by Wang *et al.* [22]. Moreover, these zinc concentration measurements show considerable variability, which is consistent with our model, both in terms of the very high flux through the free zinc compartment, and the predicted stochasticity of zinc-regulated exporter protein expression.

Although the model is able to fit the experimental data very well, two features of the model could be improved. First are the model structure and parameters for the zinc reservoir. We assume a fixed potential reservoir size, and the optimal parameters suggest that this size matches the reported concentration of zinc in the cells [40]. Moreover, the on- and off-rates for such zinc binding are poorly estimated, despite the use of informative priors. It is likely that the potential reservoir size is able to respond to the levels of zinc and that the overall size may exceed the zinc content. We have not included this in our model because of a lack of relevant experimental data. Second, we assume that the changes in growth rate between the different strains do not affect the rate of production of zinc-associated or Lux proteins. This may or may not be reasonable, and further experimentation would be appropriate.

The experimental measurements have used a Lux assay [41] to assess promoter activity. There are two areas for consideration. First, the Lux assay is an indirect measure of promoter expression and protein activity. The measured light output arises from a set of coupled chemical reactions that produce the light and recycle the substrates needed for light production [42]. Thus, although we have assumed a linear relationship between light output and protein synthesis, it is likely that this relationship is nonlinear. That said, the same plasmid lux constructs have been used previously to assess responses of several *E. coli* promoters to acid stress [43]. In that work, the lux reporters were compared with independent qPCR experiments and there was high concordance between the two reporter technologies. This gives us confidence in the reliability of the reporter results.

The second consideration is that the response of the *lux* promoter itself can be sensitive to metabolic change, and especially cell death. Indeed, Lux reporters can be used as a signal for toxicity [44]. However, such use is effective only at much higher zinc concentrations than in our study, typically greater than 100 μM in *E. coli* cells [44]. This is compatible with our own data, where no cytotoxicity was observed at 12.5 μM added zinc (figure 7*a*,*b*), some cytotoxicity at 100 μM added zinc (figure 7*b*) and more marked signal reduction at 200 μM added zinc (electronic supplementary material, figure S1*c*). Thus we are confident of our use of the lux reporter at lower concentrations of added zinc. As discussed above, a mixed cellular response at higher concentrations of zinc would suggest that future experimental and modelling approaches, that address the responses of a heterogeneous population, could be appropriate.

## 5. Material and methods

### 5.1. Bacterial strains and plasmids

Bacterial strains were constructed by the One-step inactivation method [45] and P1 transduction. PCR products were amplified with the primers: TOP631 and TOP632 (*zur* deletion); TOP1464 and TOP1465 (*zntR* deletion); TOP1468 and 1469 (*znuCB* deletion); and TOP1470 and 1471 (*zntA* deletion) (electronic supplementary material, table S2). The template plasmids: pKD13 (*zur* deletion); pKD4 (*znuCB* deletion) and pKD3 (*zntA* and *zntR* deletions) were electroporated into *E. coli* K-12 BW25113 cells containing pKD46. Kanamycin or chloramphenicol resistant clones were selected. Chromosomal gene disruption was confirmed by PCR using the specific primer for each gene (electronic supplementary material, table S2) and the common primer, k1 or c1 (described in [45]). The *Δzur*::Km*,* *ΔznuCB*::Km*,* *ΔzntA*::Cm and *zntR*::Cm mutations were transferred from *E. coli* K-12 BW25113 into *E. coli* K-12 MG1655 CGSC 7740 by P1 transduction (electronic supplementary material, table S3). Kanamycin resistance was cured from MG1655 *Δzur*::Km and MG1655 *ΔznuCB*::Km for the Lux assays, to enable the strains to be subsequently transformed with the Lux reporter plasmids, which carried the kanamycin resistance marker. This was done using FLP expressed from pCP20 [45]. The double knockout mutant of *znuCB* and *zntA* (MG1655 *ΔzntA*::Cm *ΔznuCB*) was constructed by P1 transduction of the *zntA*::Cm allele into MG1655 Δ*znuCB* cells. Luciferase reporter plasmids were constructed based on the pLUX plasmid which is a low-copy plasmid carrying the *Photorhabdus luminescens lux* operon [46]. DNA fragments (approx. 200 bp) containing promoters P*znuC* and P*zntA* were amplified with the primers, TOP1494 and 1495 (P*znuC*), TOPN01 and TOPN02 (P*zntA*), and TOP1499 and TOP1500 (P*hns*) (electronic supplementary material, table S2) and cloned into pLUX as *Xho*I/*BamH*I fragments*. Escherichia coli* K-12 DH5α cells were transformed with the plasmids, and transformants were selected on the basis of kanamycin resistance. pLUX clones containing P*znuC*, P*zntA* and P*hns* were validated by DNA sequence analysis. These plasmid constructs were subsequently introduced into the strains, MG1655, MG1655 *ΔzntA*::Cm, MG1655 *zntR*::Cm, MG1655 *Δzur*, MG1655 *ΔznuCB* and MG1655 *ΔzntA*::Cm *ΔznuCB*.

### 5.2. Lux assay

The bioluminescence reaction in bacteria involves the oxidation of reduced riboflavin phosphate (FMNH_2_), oxygen and a long chain fatty aldehyde. The *Photorhabdus luminescens lux* operon consists of *luxCDE*, encoding a fatty acid reductase complex enzyme for the synthesis of the fatty aldehyde substrate and *luxAB*, encoding the luciferase for the bioluminescent reaction [42]. Because FMNH_2_ and oxygen are present in *E. coli* cells, we could detect the bioluminescence in cells expressing the *lux* operon from zinc activated or repressed promoter/*lux* fusions without the addition of extra substrates. Pre-cultures of the various reporter plasmid carrying strains were grown overnight at 37°C, and used to inoculate fresh LB/kanamycin (at 1/500 v v^−1^) and grown for 3 h under aerobic conditions at 37°C. Two hundred microlitre aliquots of these cultures were transferred into 96-well microtitre plates (Porvair, UK). These cultures were incubated at 37°C for 10 min without shaking. Following incubation, bioluminescence was immediately measured using a GENios Pro plate luminometer (Tecan) for the static experiment. For the time course experiment, after the 3 h incubation, different amounts of ZnSO_4_ were added before the measurement of light output from the culture were taken. Twelve light output measurements were taken over 1 h at 5-min intervals. All experiments were performed using three biological replicates. For the LB experiments, we performed four replicate bioluminescence measurements for each biological replicate. To approximate the bioluminescent activity per unit cell mass, we divided the luminescence activity by the absorbance (OD_595_) of the cell culture. Thus, for all experiments, both luminescence and absorbance measurements were taken and data were collected and processed as follows: 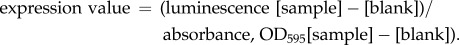


### 5.3. Bacterial growth measurements

All strains were grown in Luria Bertani broth containing varying levels of zinc sulfate as described above. Cultures of mutant strains were also supplemented with the appropriate antibiotics in order to maintain selection for the mutant. Optical density measurements were taken from each strain type in each media type in triplicate using an Ultrospec 2000 spectrophotometer (Pharmacia Biotech). Readings were taken using a 10-fold dilution and subsequent correction to maintain linearity between the estimation of cell number and observed optical density. Viable counts were taken using a variation of the Miles and Misra method [47]. Tenfold serial dilutions were made from individual cultures and triplicate 5 μl spots were placed onto prewarmed LB-agar plates. These were incubated for 12–16 h at 37°C, and colony numbers were recorded using dilutions that gave between 20 and 100 individually discernable colonies. Viable counts were calculated on the basis of the appropriate corrections for dilution factor and 5 μl spot size.

### 5.4. Zinc concentration in LB

The zinc concentration of the LB media used in these experiments was analysed using Thermo-Fisher Scientific X-Series II inductively coupled mass spectrometry (ICP-MS). Samples were applied via an autosampler (Cetac ASX-520) through a concentric glass nebulizer (Thermo-Fisher Scientific) at a rate of 1 ml min^−1^. Interfering ions were removed by the hexapole collision cell (7% hydrogen in helium) and data analysed using Plasmalab software (v. 2.5.4; Thermo-Fisher Scientific).

### 5.5. Monte Carlo simulations

Parameter estimations were carried out using the Metropolis–Hastings algorithm [29,30]. For the majority of parameters, uninformative priors have been used. Informative priors have been used for the parameters *l*_1_, *m*_1_, *K*_Ron_, *K*_Roff_ and the six *γ* parameters (electronic supplementary material, table S4). For the growth rate parameters, prior distributions were based on the growth rates of strains grown in LB. For each growth curve, the Richards model [47] was fitted to estimate the growth rate. The mean rate for each strain was computed, along with the mean standard deviation of all strains, to provide a Gaussian prior for each strain. As proposal distributions, a lognormal distribution was used and the variances of the distributions were empirically chosen in order to ensure acceptance probabilities close to 0.234 [48]. The parameters were updated separately in each step, and 4 000 000 iterations were carried out. To calculate the steady states of the system, Newton algorithm of GSL library [49] encoded in C++ was applied. ODE calculations were performed by the cvode solver with Newton iterations provided by the Sundials library [50].

### 5.6. Calculations of dynamical and stochastic simulations

For model simulations with a specific parameter value set, ODE calculations were performed by the deSolve package [51] in the statistical environment *R*. Stochastic simulations based on the Gibson–Bruck algorithm [31] were performed until 1 × 10^6^ s, i.e. 277 h, using Dizzy (v. 1.11.3) [52] using 29 chemical reactions including 14 species (the reaction scheme is given in the supporting SBML file zinc.xml).

## Supplementary Material

Figure S1

## Supplementary Material

Figure S2

## Supplementary Material

Figure S3

## Supplementary Material

Figure S4

## Supplementary Material

Figure S5

## Supplementary Material

Tables_S1_S2_S3_S4.doc

## Data accesability

Experimental data are available on Figshare (http://dx.doi.org/10.6084/m9.figshare.1311513). Mathematical models are available on Biomodels (MODEL1502180000).
