# Supplementary figures and images for: The dynamic balance of import and export of zinc in Escherichia coli suggests a heterogeneous population response to stress
Source: J R Soc Interface. 2015 May 6;12(106):20150069. doi: 10.1098/rsif.2015.0069 (PMC4424684; doi:10.1098/rsif.2015.0069)

### LB

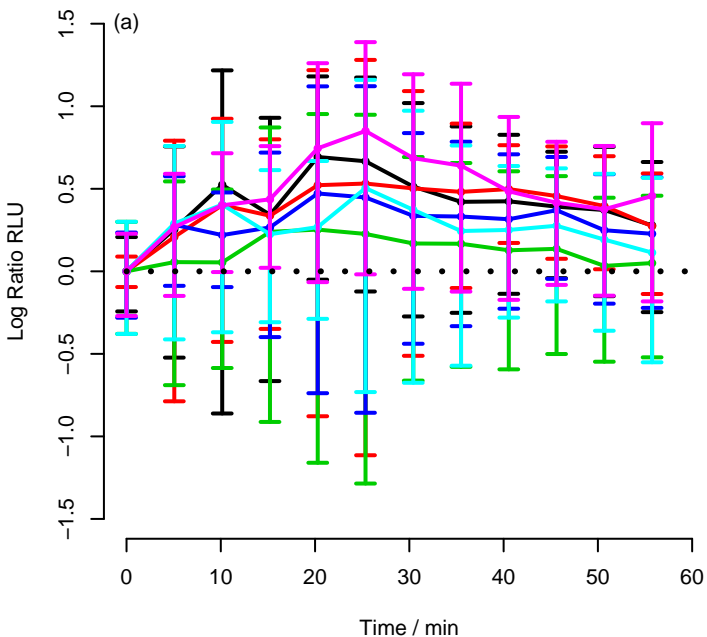

### LB + 100uM Zn

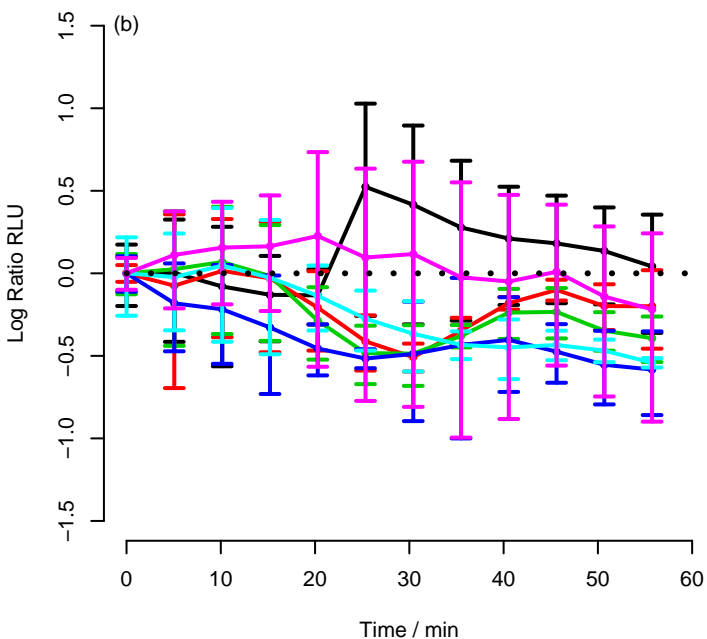

### LB + 200uM Zn

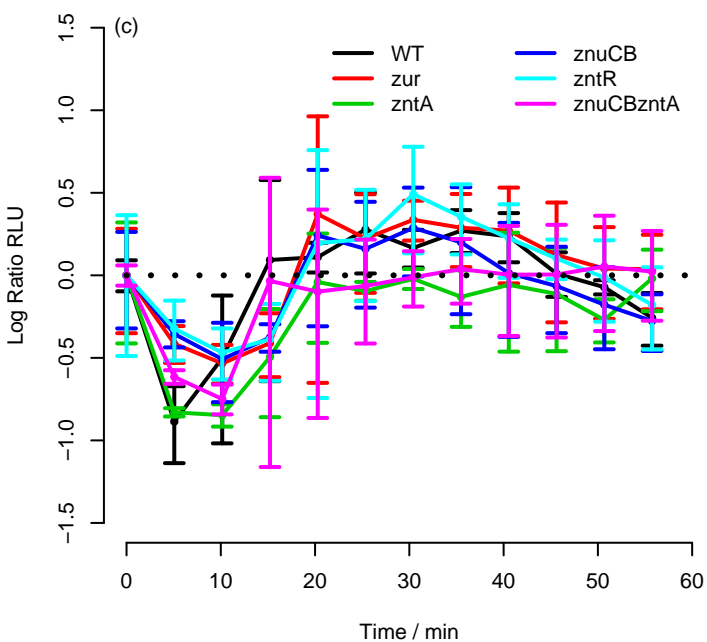

Supplement: Figure S1 [file rsif20150069supp1.pdf]

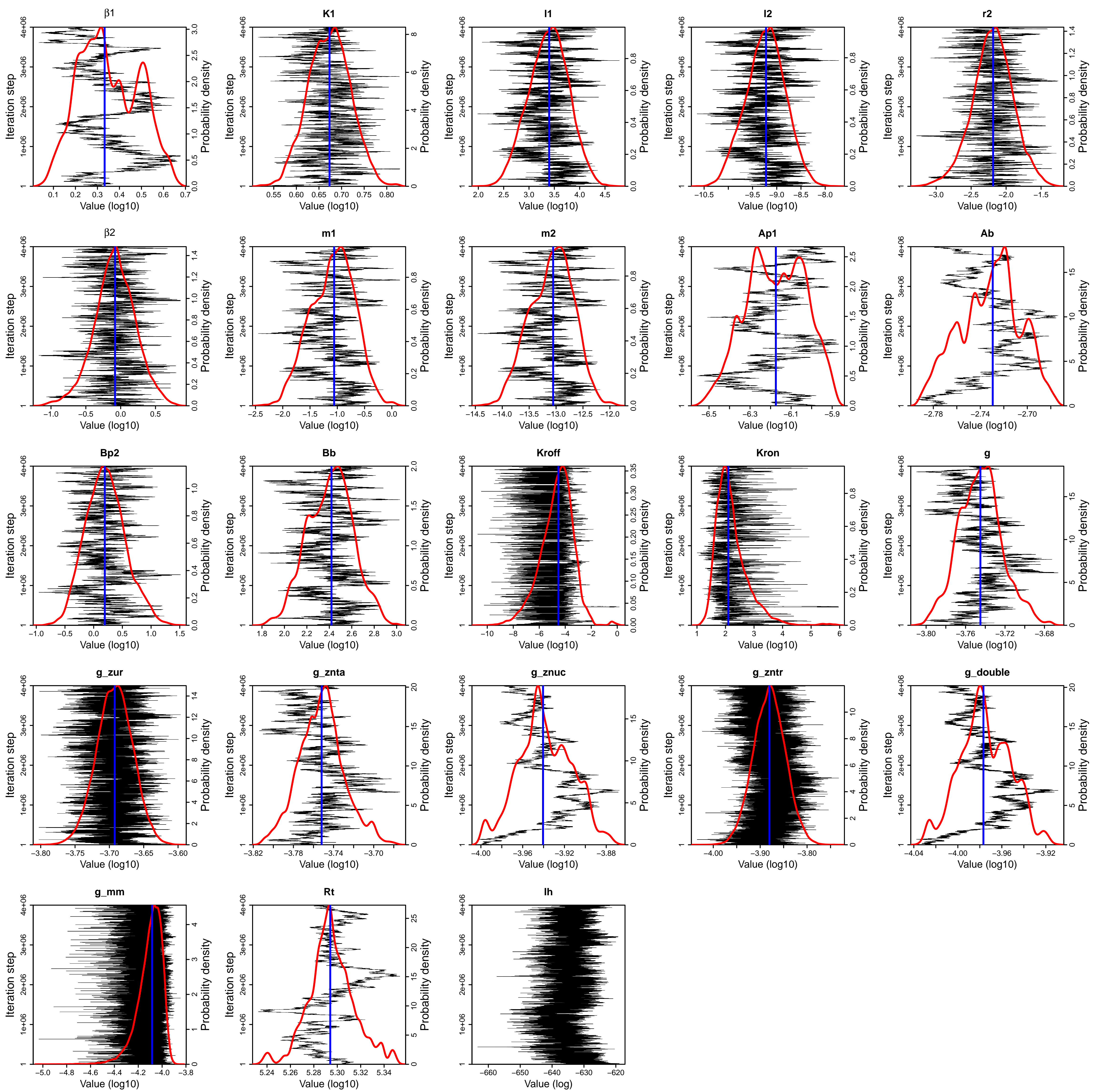

Supplement: Figure S3 [file rsif20150069supp3.pdf]
