# Supplementary material for: The dynamic balance of import and export of zinc in Escherichia coli suggests a heterogeneous population response to stress
Source: J R Soc Interface. 2015 May 6;12(106):20150069. doi: 10.1098/rsif.2015.0069 (PMC4424684; doi:10.1098/rsif.2015.0069)
Supplement: Figure S2 [file rsif20150069supp2.pdf]

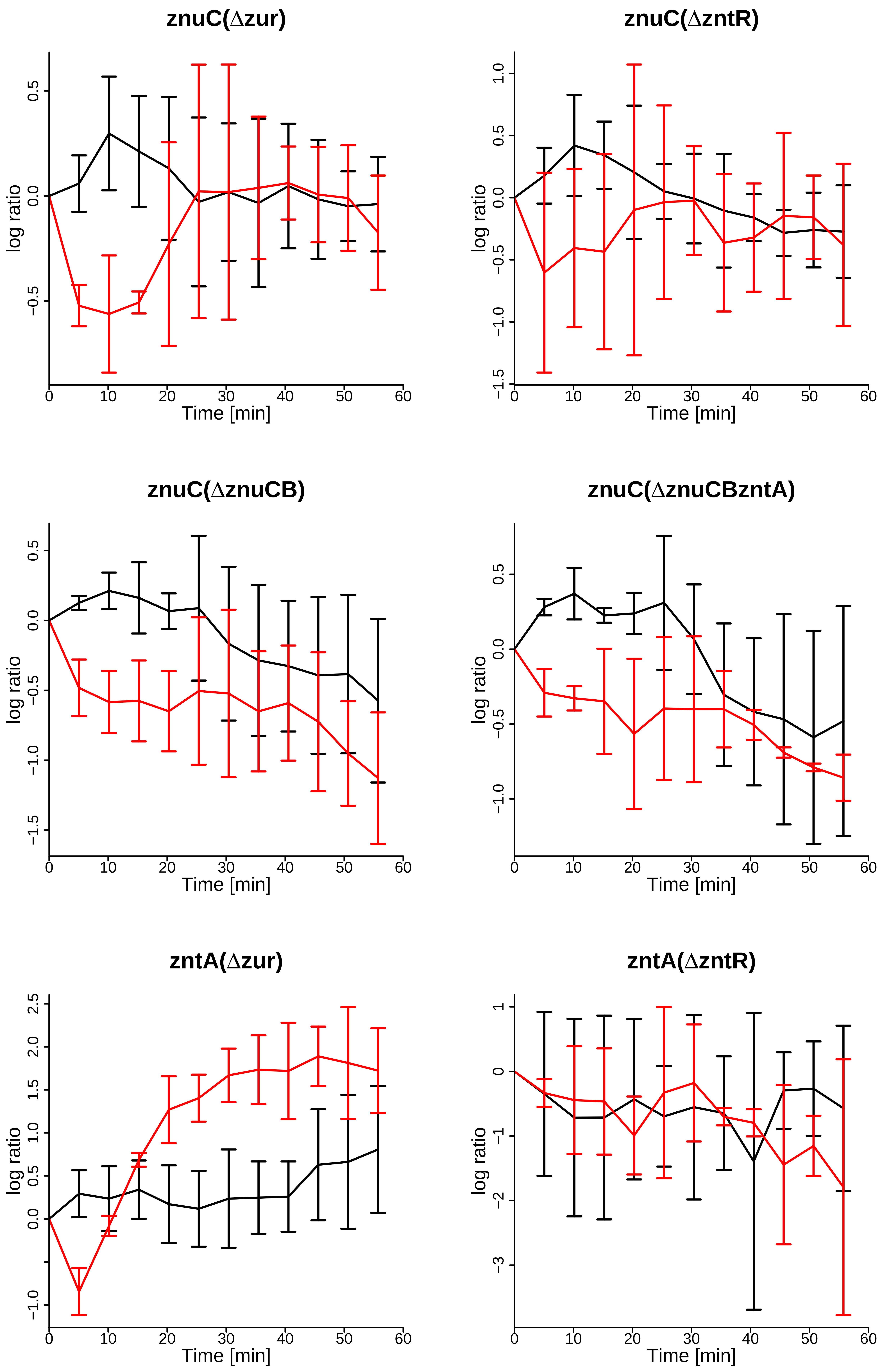
znuC(Δzur)
log ratio
0.5
0.0
−0.5
0
10
20
30
40
50
60
Time [min]
znuC(ΔzntR)
1.0
0.5
0.0
−0.5
−1.0
−1.5
znuC(ΔznuCB)
−1.0
−1.5
znuC(ΔznuCBzntA)
zntA(Δzur)
2.5
2.0
1.5
1.0
zntA(ΔzntR)
1
0
−1
−2
−3
