# Supplementary material for: The dynamic balance of import and export of zinc in Escherichia coli suggests a heterogeneous population response to stress
Source: J R Soc Interface. 2015 May 6;12(106):20150069. doi: 10.1098/rsif.2015.0069 (PMC4424684; doi:10.1098/rsif.2015.0069)
Supplement: Figure S4 [file rsif20150069supp4.pdf]

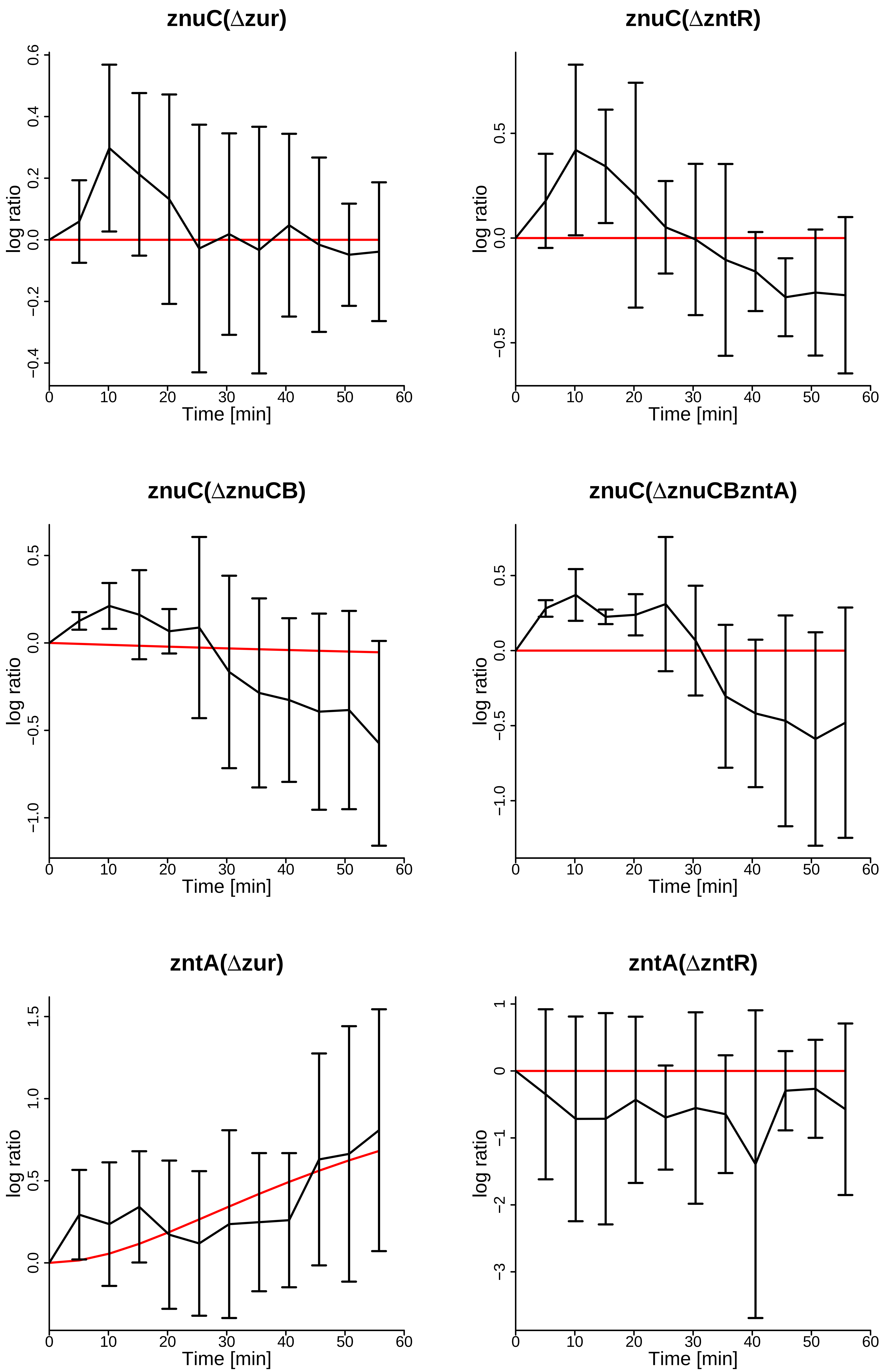
znuC(Δzur)
znuC(ΔzntR)
znuC(ΔznuCB)
znuC(ΔznuCBzntA)
zntA(Δzur)
zntA(ΔzntR)
log ratio
Time [min]
