# Supplementary material for: The dynamic balance of import and export of zinc in Escherichia coli suggests a heterogeneous population response to stress
Source: J R Soc Interface. 2015 May 6;12(106):20150069. doi: 10.1098/rsif.2015.0069 (PMC4424684; doi:10.1098/rsif.2015.0069)
Supplement: Figure S5 [file rsif20150069supp5.pdf]

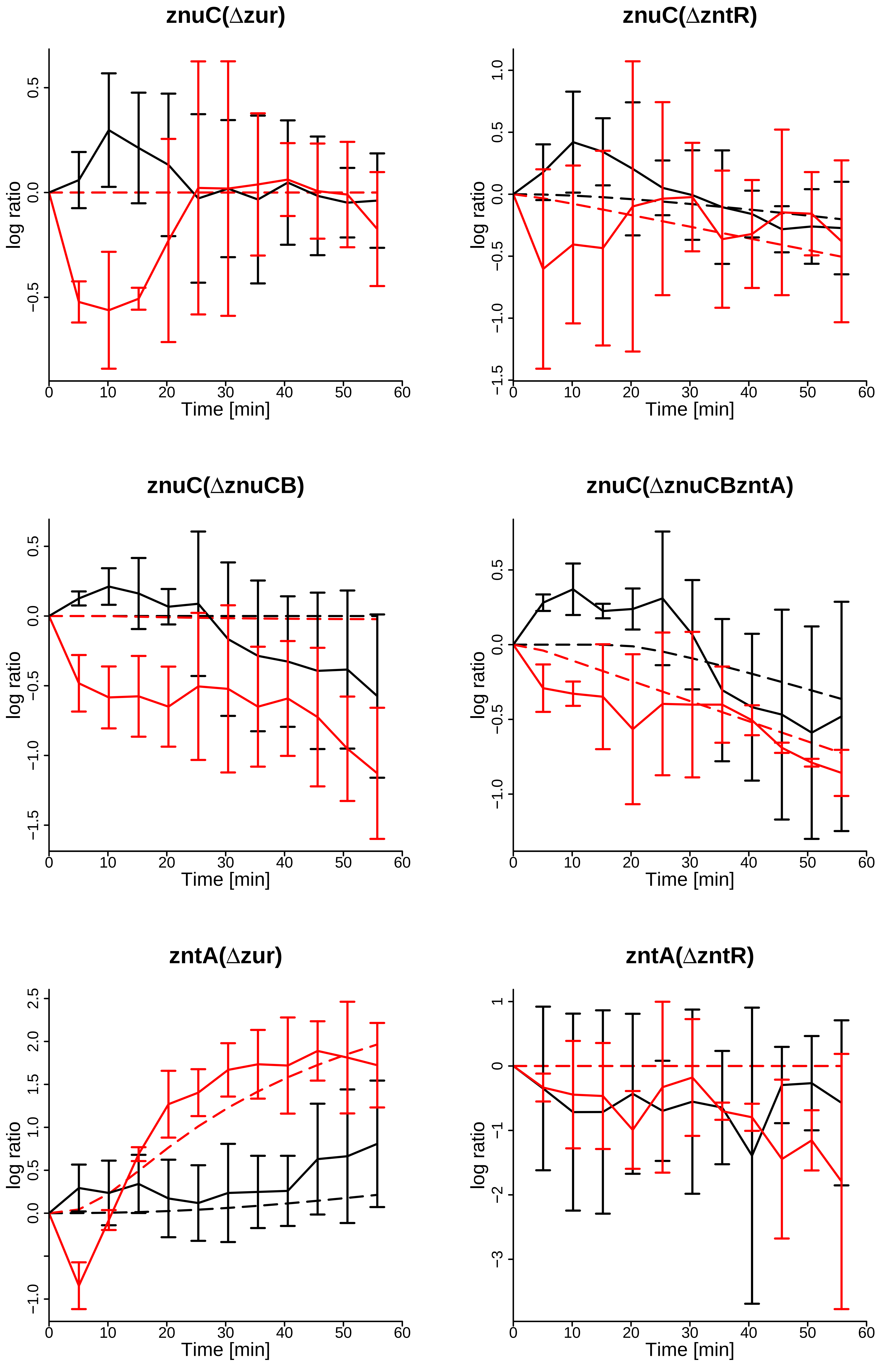

znuC(Δzur)
znuC(ΔzntR)
znuC(ΔznuCB)
znuC(ΔznuCBzntA)
zntA(Δzur)
zntA(ΔzntR)
log ratio
Time [min]
0 10 20 30 40 50 60
0.5 0.0 −0.5
1.0 0.5 0.0 −0.5 −1.0 −1.5
0.5 0.0 −0.5 −1.0 −1.5
0.5 0.0 −0.5 −1.0
2.5 2.0 1.5 1.0 0.5 0.0 −1.0
1 0 −1 −2 −3
