# Supplementary material for: The dynamic balance of import and export of zinc in Escherichia coli suggests a heterogeneous population response to stress
Source: J R Soc Interface. 2015 May 6;12(106):20150069. doi: 10.1098/rsif.2015.0069 (PMC4424684; doi:10.1098/rsif.2015.0069)
Supplement: Tables_S1_S2_S3_S4.doc [file rsif20150069supp6.doc]

**Table S1: Estimates of all model parameters**

| **Parameter** | **Median** | **Coefficient of variation** | **Lower bound** | **Upper bound** | **Units** |
| --- | --- | --- | --- | --- | --- |
| β_1_ | 2.15 | 0.317 | 1.17 | 3.95 | nMs^-1^ |
| K_1_ | 4.71 | 0.107 | 3.82 | 5.81 | nM |
| l_1_ | 2.50×10^3^ | 1.12 | 4.25×10^2^ | 1.45×10^4^ | nM^-2^s^-1^ |
| l_2_ | 6.01×10^-10^ | 1.17 | 9.67×10^-11^ | 3.70×10^-9^ | s^-1^ |
| r_2_ | 6.52×10^-3^ | 0.754 | 1.74×10^-3^ | 2.43×10^-2^ | nMs^-1^ |
| β_2_ | 0.830 | 0.774 | 0.215 | 3.18 | s^-1^ |
| m_1_ | 8.75×10^-2^ | 1.16 | 1.43×10^-2^ | 0.532 | nM^-2^s^-1^ |
| m_2_ | 8.82×10^-14^ | 1.16 | 1.43×10^-14^ | 5.41×10^-13^ | nMs^-1^ |
| A_P1_ | 6.69×10^-7^ | 0.336 | 3.52×10^-7^ | 1.27×10^-6^ | nM^-1^s^-1^ |
| A_b_ | 1.86×10^-3^ | 0.0587 | 1.66×10^-3^ | 2.09×10^-3^ | s^-1^ |
| B_P2_ | 1.56 | 0.951 | 0.322 | 7.55 | nM^-1^s^-1^ |
| B_b_ | 2.63×10^2^ | 0.490 | 1.05×10^2^ | 6.51×10^2^ | s^-1^ |
| K_Roff_ | 2.99×10^-5^ | 63.8 | 1.02×10^-7^ | 8.49×10^-3^ | s^-1^ |
| K_Ron_ | 1.25×10^2^ | 2.23 | 9.02 | 1.72×10^3^ | nM^-1^s^-1^ |
| γ_WT_ | 1.80×10^-4^ | 0.0512 | 1.63×10^-4^ | 1.99×10^-4^ | s^-1^ |
| γ_zur_ | 2.03×10^-4^ | 0.0603 | 1.80×10^-4^ | 2.29×10^-4^ | s^-1^ |
| γ_zntA_ | 1.77×10^-4^ | 0.0519 | 1.60×10^-4^ | 1.96×10^-4^ | s^-1^ |
| γ_znuC_ | 1.15×10^-4^ | 0.0591 | 1.02×10^-4^ | 1.29×10^-4^ | s^-1^ |
| γ_zntR_ | 1.32×10^-4^ | 0.0786 | 1.13×10^-4^ | 1.54×10^-4^ | s^-1^ |
| γ_znuCzntA_ | 1.05×10^-4^ | 0.0533 | 9.50×10^-5^ | 1.17×10^-4^ | s^-1^ |
| γ_MM_ | 8.23×10^-5^ | 0.241 | 5.15×10^-5^ | 1.31×10^-4^ | s^-1^ |
| R_T_ | 1.97×10^5^ | 0.0454 | 1.80×10^5^ | 2.15×10^5^ | nM |

All model parameters have been estimated from the posterior distributions of the MCMC runs. We report here: the estimated mean value for each parameter, the coefficient of variation and lower and upper bounds, that have been calculated by subtracting or adding 1.96 times the standard deviation from the median log value: this process, assuming a Gaussian posterior centered on the median value, has been carried out to avoid sampling errors at the extreme values of the Markov chain. The majority of the parameters can be estimated from the data with reasonable precision, but some parameters still have some considerable variation.

**Table S2: Primers used for cloning**

| **Primer** | **Sequence** | **Purpose** |
| --- | --- | --- |
| TOP631 | ctcaatgtttttcctggaacatggtaaagtaaggacattcttaaccccactttgaggtgattccggggatccgtcgacc | construction of *zur* deletion |
| TOP632 | caatgaatatcgctggtaattaatccctcctgcccgacgtgtacaaggctgtacgccctcgtgtaggctggagctgcttcg | *zur* deletion |
| TOP1464 | tacgcggcgattatgctgacaagttgttggacaaaatcaacgataactagtggagtatgttgtgtaggctggagctgcttc | *zntR* deletion |
| TOP1465 | ctcataaaaactccagttggttatttaacggcgcgagtgtaatcctgccagtgcaaaaaacatatgaatatcctccttag | *zntR* deletion |
| TOP1468 | aatgaatatgagaagtgtgatattataacatttcatgactactgcaagactaaaattaactgtgtaggctggagctgcttc | *znuCB* deletion |
| TOP1469 | ccgaaccgtaggtcggataaggcgctcgcgccgcatccgacaaatgtgttcagcgataga catatgaatatcctccttag | *znuCB* deletion |
| TOP1470 | tggagtcgactccagagtgtatccttcggttaatgagaaaaaacttaaccggaggatgcctgtgtaggctggagctgcttc | *zntA* deletion |
| TOP1471 | taaccctctccccagaggggcgaggggaccgatcgcgctcaatgttgcgatcggtttgcccatatgaatatcctccttag | *zntA* deletion |
| TOP1401 | cccgcaatgaatatcgctggtaat | check *zur* deletion |
| TOP1480 | tgagcaacggagagaagcaattcag | check *zntR* deletion |
| TOP1482 | gaagcgatgaacccaacgggtttaag | check *znuCB* deletion |
| TOP1483 | gctgtttatcagtaactttgtctggc | check *zntA* deletion |
| TOP1494 | ccgctcgaggcagaagcgatgaacccaacggg | cloning of *znuC* promoter |
| TOP1495 | cgcggatcctgcagtagtcatgaaatgttataatatc | *znuC* promoter |
| TOPN01 | ccgctcgagagtaactttgtctggctggggagcc | *zntA* promoter |
| TOPN02 | cgcggatcctaagttttttctcattaaccgaagg | *zntA* promoter |
| TOP1499 | ccgctcgagctcacgtgctgcgaaatcatcggtg | *hns* promoter |
| TOP1500 | cgcggatcccttatattggggtggtttgttgagg | *hns* promoter |

**Table S3: Strain and Plasmid Lists**

| **Name** | **Relevant Genotype** | **Source and/or Reference** |
| --- | --- | --- |
| **Strains** | | |
| DH5α | *supE*44 Δ*lacU*169(ø80 *lacZ*ΔM15) *hsdR*17 *recA*1 *endA*1 *gyrA*96 *thi*-1 *relA*1 |  |
| BW25113 | *lacI^q^ rrnB*_T14_ Δ*lacZ*_WJ16_ *hsdR514* Δ*araBAD*_AH33_ Δ*rhaBAD*_LD78_ | Datsenko and Wanner 2000 (PNAS, vol 97, 6640-6645) |
| MG1655 CGSC 7740 | prototroph | Lab stock |
| TON2367 | MG1655(CGSC7740) Δ*zntR::Cm* | This work |
| TON2365 | MG1655(CGSC7740) Δ*zntA::Cm* | This work |
| TON2383 | MG1655(CGSC7740) Δ*zur*(-)^a^ | This work |
| TON2377 | MG1655(CGSC7740) Δ*znuCB*(-)^a^ | This work |
| TON2447 | MG1655(CGSC7740) Δ*znuCB*(-) Δ*zntA::Cm,* The *ΔzntA::Cm* allele was transduced in TON 2383 by P1 transduction. | This work |
| **Plasmids** | | |
| pLUX | parental plasmid | Burton et al., 2010 (JMB, vol 401, 726-742) |
| pLUX*znuC* | From -200 to -15 in the upstream of znuC gene are cloned at *Xho*I-*BamH*I site in pLUX.^b^ | This study |
| pLUX*zntA* | From -190 to -14 in the upstream of znuC are cloned at *Xho*I-*BamH*I site in pLUX.^b^ | This study |
| pLUX*hns* | DNA fragment from -200 to -16 relative to *hns* start codon are cloned at *Xho*I-*BamH*I site in pLUX.^b^ | This study |

| a: Km cassette was removed by the expression of FLP supplied by pCP20. See detail in Datsenko and Wanner 2000 (PNAS, vol97, 6640-6645) |
| --- |
| b: The start and end positions of fragments cloned in pLUX are relative to the first nucelotide of an initiation codon in each gene. |

**Table S4: Informative Prior Distributions**

| **Parameter** | **As Variable** | **Mean** | **s.d.** |
| --- | --- | --- | --- |
| l_1_ | log10(x) | 1.30 | 0.5 |
| m_1_ | log10(x) | 1.28 | 0.5 |
| K_Ron_ | log10(x) | 1.27 | 1 |
| K_Roff_ | log10(x) | -2.72 | 1.97 |
| γ_WT_ | x*3600 | 0.667 | 0.073 |
| γ_zur_ | x*3600 | 0.437 | 0.073 |
| γ_zntA_ | x*3600 | 0.448 | 0.073 |
| γ_znuC_ | x*3600 | 0.593 | 0.073 |
| γ_zntR_ | x*3600 | 0.690 | 0.073 |
| γ_znuCzntA_ | x*3600 | 0.541 | 0.073 |
| γ_MM_ | x*3600 | 0.334 | 0.073 |

For the majority of variables, uninformative prior distributions were used. Informative priors were used for the variables listed in this table. For l_1_, m_1_, K_Roff_ and K_Ron_, the medians were based on theoretical considerations and then given broad standard deviations. According to the classical theory of reaction kinetics in solution for a diffusion limited reaction (Moore WJ 1972 *Physical Chemistry*, 4th ed. Prentice Hall, Englewood Cliffs), the reaction rate *β* between two chemical species with diffusivities D_1_ and D_2_ is: β=4πd_12_(D_1_+D_2_) where *d_12_* is the reaction diameter, i.e., sum of the radii of both species. The diffusivity of a particle of radius *r*, at absolute temperature *T*, in a medium with viscosity *η* is D=kT/6πr*η* where k is the Boltzmann constant (Barrow GM 1996 *Physical Chemistry*. Mcgraw-Hill College). For a spherical protein, r can be calculated as r=0.066M^1/3^ where M is the mass of the protein in Daltons (Erickson HP 2009 *Biol Proced Online* **11:**32-51). The median of the prior binding rates l_1_ and m_1_ were calculated using these equations, with the mass of Zur at 19.254kDa (<http://www.uniprot.org/uniprot/P0AC51>), ZntR at 16.179kDa (<http://www.uniprot.org/uniprot/P0ACS5>), and the diameter of Zn(II) at 74pm (<http://www.webelements.com/zinc/atom_sizes.html>) and taking diffusion in cytoplasm as approximately four times slower than in water (Verkman AS 2002 *Trends Biochem Sci* **27:**27-33) together with a generous standard deviation. Similar calculations were used for the reservoir parameters. For *K_Ron_*, a median value was calculated assuming a protein mass of 31.26kDa, that being the median value of all proteins in *E. coli* (<http://www.bioinformatics.org/sms2/protein_mw.html>). For *K_Roff_*, we took the mean and standard deviaton of the affinities of zinc finger domain (Table 6 of Dudev T, Lim C 2003 *Chem Rev* **103:**773-788), Zur, and ZntR (Table 2 in Cui et al. 2008). These give very broad prior estimates for these parameters. For the gamma parameters, these priors were based on experimental measurements of the growth rates using optical density and so are much more tightly defined (see *Materials and Methods)*.
